# YBX1 Is a Modulator of MIA/CD-RAP-Dependent Chondrogenesis

**DOI:** 10.1371/journal.pone.0082166

**Published:** 2013-12-12

**Authors:** Rainer Schmid, Katharina Meyer, Rainer Spang, Birgit Schittek, Anja Katrin Bosserhoff

**Affiliations:** 1 Institute of Pathology, University of Regensburg, Regensburg, Germany; 2 Institute for Functional Genomics, University of Regensburg, Regensburg, Germany; 3 Institute of Dermatology, University of Tübingen, Tübingen, Germany; University of Tennessee, United States of America

## Abstract

MIA/CD-RAP is a small, secreted protein involved in cartilage differentiation and melanoma progression. We recently revealed that p54^nrb^ acts as a mediator of MIA/CD-RAP action to promote chondrogenesis and the progression of malignant melanoma. As the molecular mechanism of MIA/CD-RAP action in cartilage has not been defined in detail until now, we aimed to understand the regulation of p54^nrb^ transcription in chondrogenesis. We concentrated on the previously described MIA/CD-RAP-dependent regulatory region in the *p54^nrb^* promoter and characterized the transcriptional regulation of p54^nrb^ by MIA/CD-RAP in cartilage. A series of truncated *p54^nrb^* promoter constructs and mutagenesis analysis revealed that the transcription factor YBX1, which has not been investigated in chondrogenesis thus far, is the mediator of MIA/CD-RAP dependent activation of p54^nrb^ transcription. A systematic analysis of genes carrying this binding site in their promoter region revealed further potential MIA/CD-RAP-regulated genes that have been implicated in cartilage differentiation. In summary, we described the effects of MIA/CD-RAP on transcriptional regulation in chondrocytes. Understanding the regulation of p54^nrb^ via YBX1 contributes to the understanding of chondrogenesis. Uncovering new downstream effectors that function via the activation of YBX1 supports the important role of MIA/CD-RAP in these processes.

## Introduction

Melanoma inhibitory activity (MIA)/cartilage-derived retinoic acid-sensitive protein (CD-RAP) is secreted from malignant melanoma cells [1], [2] as well as chondrocytes [3]. Developmental expression analysis by *in situ* hybridization of mouse embryos shows that MIA/CD-RAP expression is initiated at the beginning of chondrogenesis and is subsequently restricted to cartilage tissue, in which MIA/CD-RAP expression remains abundant throughout development [4], [3]. MIA/CD-RAP expression is tightly correlated to the expression of cartilage specific type II collagen [4] and to the expression of the chondrogenic differentiation marker aggrecan during cartilage development [5]. Analysis of the role of MIA/CD-RAP during cartilage development revealed that MIA/CD-RAP by itself is not capable to induce the differentiation of mesenchymal stem cells. However, together with bone morphogenetic protein (BMP)-2 and transforming growth factor (TGF)-β3, MIA/CD-RAP enhances the chondrogenic phenotype while inhibiting osteogenic differentiation during mesenchymal stem cell differentiation [6].

Recent expression analysis in cartilage tissue derived from MIA/CD-RAP-deficient mice revealed a strong down-regulation of p54^nrb^ (non-POU-domain-containing octamer-binding protein) [7]. The p54^nrb^ protein was first isolated from the cervical cell line HeLa [8] and has nucleic acid binding abilities that enable its dual roles in transcription and splicing [8], [9]. It is known that p54^nrb^ retains messenger RNAs in the nucleus and therefore influences gene expression [10]. In chondrogenesis, p54^nrb^ interacts with the transcription factor Sox9 and enhances the Sox9-mediated activation of the *Col2a1* promoter promoting chondrogenesis [11]. We were able to show that p54^nrb^ acts as a mediator of MIA/CD-RAP action to promote chondrogenesis through the inhibition of proliferation by negative cell cycle regulation via Cyclin D2 and through the induction of differentiation via enhanced Sox9-dependent *COL2A1* promoter activity [7].

We also found strongly down-regulated p54^nrb^ protein levels [7] and decreased *p54^nrb^* promoter activity [12] in a previously described MIA-deficient HMB2 melanoma cell system [13], [14]. As an MIA/CD-RAP target molecule, p54^nrb^ is involved in the development and progression of malignant melanoma [12]. Because melanoma cell lines have been shown to have strong *p54^nrb^* promoter activity, the regulation of p54^nrb^ via MIA/CD-RAP was shown to involve transcriptional regulation. Recently, we identified one highly conserved region in the p54^nrb^ promoter that is necessary for MIA-dependent activation in melanoma [15]. Additionally, we defined the transcription factor Y-box binding protein 1 (YBX1) as the mediator of MIA/CD-RAP dependent p54^nrb^ transcription [15]. YBX1 is a multifunctional protein, which regulates transcription through binding to promoters containing the Y-box motif (inverted CCAAT-Box) [16], [17] and inhibits translation through masking of mRNA or regulation of mRNA stability [18], [19]. *In vivo* YBX1 is expressed throughout embryonal development and its expression correlates with cell proliferation [20]. The majority of YBX1-*knockout* embryos develop normally until day 13.5 of development. Afterwards severe growth retardation and mortality was observed, uncovering a nonredundant role of YBX1 in late stages of embryonic development [21].

The aim of this study was to characterize the transcriptional regulation of p54^nrb^ by MIA/CD-RAP during cartilage development to explain the action of MIA/CD-RAP during this process. To examine p54^nrb^ transcription during chondrogenesis, we used primary murine mesenchymal stem cells isolated from MIA/CD-RAP-deficient mice and analyzed the already known highly conserved MIA/CD-RAP-regulated *p54^nrb^* promoter region.

## Materials and Methods

### Animals

Transgenic mice were generated as previously described [22]. C57Bl/6 control and MIA/CD-RAP knockout mice were bred under specified pathogen-free conditions at 26°C and 70% relative humidity and were kept under a 12 hour light/12 hour dark cycle at the University of Regensburg. They were fed a breeding/maintenance diet (Altromin GmbH, Lage, Germany) and given water *ad libitum*. The mice were randomly housed in polypropylene cages with sawdust bedding. The cages were sanitized twice each week. Animal care and all experimental procedures were carried out in accordance with the guidelines of the German law governing animal care and use in biomedical research. All efforts were made to minimize the number of animals used and their suffering. Adult mice were sacrificed via cervical dislocation after anesthetization by isoflurane (2-chloro-2-(difluoromethoxy)-1,1,1-trifluoro-ethane) inhalation. According to the German Animal Welfare Act 2006 (article 4), it is sufficient to obtain supervision from the local animal welfare officer (Dr. Thilo Spruss, University Hospital Regensburg) for the killing of mice for scientific purposes (including tissue, embryo and cell extraction) if no experimental procedures were carried out in the animals. As this was the case in this study, no further notification or approval by the Ethics Committee for Animal Research of the Bavarian government was necessary.

### Human tissue samples

The sampling and handling of human tissue was carried out in accordance with the ethical principles of the Declaration of Helsinki and was approved by the ethical committee of the University of Regensburg. As no information about the patients was used, other than the type of tumor, no written informed consent for the use of the samples in research was deemed necessary by the ethical committee.

### Cell isolation and cell culture of primary murine mesenchymal stem cells (mMSCs)

Murine MSCs were isolated and cultured as previously described [7]. All experiments were performed in cells between passages three and six.

### Differentiation

The differentiation of mMSCs was performed in two-dimensional (2D) cultures. For this procedure, the cells were cultured in induction medium including DMEM (PAA Laboratories Inc., Dartmouth, NH, USA), 0.3% glucose (Sigma-Aldrich Corp., St. Louis, MO, USA), 0.4% MEM vitamins (Life Technologies Inc., Carlsbad, CA, USA), 100 U/ml penicillin, 100 µg/ml streptomycin (both PAA), 500 ng/ml amphotericin-B, 0.1 µM dexamethasone, 1 mM sodium pyruvate, 0.17 mM ascorbic acid-2-phosphate, 0.35 mM proline (all Sigma), 5 µg/ml insulin, 5 µg transferrin, 5 ng selenous acid (ITS™ Premix; Becton Dickinson Biosciences, San Jose, CA, USA) and 10 ng/mL human TGF-β3 (HumanZyme Inc., Chicago, IL, USA). Each experiment was conducted with at least three different lots of cells. Cells were harvested for isolation of RNA and protein.

### Luciferase assay

For transient transfections, 1 – 1.5×10^5^ mMSCs (per well) were seeded into 6-well plates and transfected with 0.5 µg of plasmid DNA using the Lipofectamine Plus method (Life Technologies Inc.) according to the manufacturer's instructions. Murine MSCs were cultured in induction medium (see above) containing 10 ng/mL human TGF-β3 (HumanZyme Inc.) to induce chondrogenic differentiation. The cells were lysed 24 h after transfection, and the luciferase activity in the lysate was quantified with a luminometer using the Dual-Luciferase Reporter Assay System (Promega, Mannheim, Germany). Transfection efficiency was normalized according to the *Renilla* luciferase activity produced by cotransfection of 0.1 µg of the pRL-TK plasmid (Promega). Basal activity resulting from the pGL3basic or pGL4.10[*luc2*] vector was set to 1. All transfections were repeated at least twice.

### Luciferase reporter constructs

For transient transfection, the YBX1 promoter reporter construct (YBX1Luc) was kindly provided by Per S. Holm [23]. The YBX1 II reporter construct (YBX1Luc II) was kindly provided by Kiyoshi Higashi. Four copies of the Y-box consensus oligo (CTGATTGGCTAA) linked to a minimal promoter containing only a TATA box were cloned into the pGL3basic vector [24]. The human (hp54^nrb^Luc) and mouse (mp54^nrb^Luc) p54^nrb^ promoter reporter constructs, as well as the respective mutated and deleted hp54^nrb^Luc fragments, were used as previously described [15]. The human *p54^nrb^* construct spans from −8436 to −6833 bp, and the mouse *p54^nrb^* construct spans from −9160 to −7461 bp, with the numbering of each fragment relative to the translation start site of the gene.

### siRNA transfection

The siRNAs against mouse MIA/CD-RAP (Mm_MIA 20-43; see Table 1 for all sequences) were purchased from Sigma. Control siRNA was synthesized by Qiagen (Hilden, Germany). One day after seeding mMSCs into 6-well plates (1×10^5^ per well), the cells were transfected with siRNA using the Lipofectamine 2000 reagent (Life Technologies Inc.) according to the manufacturer's specifications. One day after siRNA transfection, the cells were transiently transfected with plasmid DNA for the luciferase assay as described above. The knockdown efficiency was assessed using quantitative real-time PCR (see below).

**Table 1:** Oligonucleotides used in the study as indicated in Material and Methods.

Name	Sequence
siRNA mMIA/CD-RAP	sense strand: 5′-CAGGUUUCCUUGAUAUUCAGC-3′
	antisense strand: 5′-GCUGAAUAUCAAGGAAACCUG-3′
siRNA control	sense strand: 5′-UUCUCCGAACGUGUCACGU-3′
	antisense strand: 5′-ACGUGACACGUUCGGAGAA-3′
5′-hp54^nrb^ (EMSA)	5′-TCTGGCCTTGATTGGCAGTTTAG-3′
3′-hp54^nrb^ (EMSA)	5′-CTAACCAATCGAGAACGCCATTTT-3′
5′-hp54^nrb^ Mut I (EMSA)	5′-TCTGGCCTTGATATCCAGTTTAG-3′
5′-hp54^nrb^ Mut II (EMSA)	5′-TCTGGCCTTCCATGGCAGTTTAG-3′

### RNA isolation and reverse transcription-PCR

Total RNA was isolated from cultured cells using the e.Z.N.A. ® MicroElute® Total RNA Kit (Omega Bio-Tek, Norcross, GA, USA) according to the manufacturer's protocol. RNA concentration and purity was measured using a NanoDrop spectrophotometer (peqlab Biotechnologie GmbH, Erlangen, Germany). Complementary DNA (cDNA) was generated by the reverse transcription of 500 ng of total RNA as previously described [12].

### Quantitative Real-Time PCR (qRT-PCR)

Quantitative RT-PCR analysis of murine MIA/CD-RAP and Gnas mRNA expression was performed using a LightCycler® 480 system (Roche, Mannheim, Germany) as described elsewhere [25]. The primers used for PCR were obtained from Sigma and are shown in Table 2. Each analysis was performed in at least duplicate. The expression ratios of the analyzed genes were calculated using an internal control and a standard curve of β-actin levels.

**Table 2:** Primers used for quantitative real-time PCR (qRT-PCR).

Gene	Species	Sequence (forward/reverse)	Product size [bp]	NCBI reference sequence
*Actb*	mouse	5′-TGGAATCCTGTGGCATCCATGAAAC-3′	320	NM_007393
		5′-TAAAACGCAGCTCAGTAACAGTCCG-3′		
*Gnas*	mouse	5′-GCAGAAGGACAAGCAGGTCT-3′	126	NM_201616
		5′-CCCTCTCCGTTAAACCCATT-3′		
*Mia1*	mouse	5′-CCAAGCTGGCTGACTGGAAG-3′	207	NM_019394
		5′-GCCAGGTCTCATAGTAACC-3′		

### Western blot analysis

Western blot analysis was performed as previously described [12]. After blotting onto PVDF membranes (Bio-Rad, Richmond, CA, USA) and blocking for 1 h with 3% BSA/PBS (p54^nrb^) or 5% milk/TBST (0.1%) (phospho-YBX1), the membranes were incubated with one of the following antibodies: anti-p54^nrb^ (1∶2000; BD Biosciences, Bedford, MA, USA), anti-phospho-YB1 (Ser102) (1∶500; #2900, Cell Signaling, Danvers, MA, USA) or anti-β-actin (Sigma).

### Preparation of nuclear extracts

Nuclear extracts were prepared from cultured cells using the method described by Dignam et al. [26]. For the isolation of nuclear extracts, murine mesenchymal stem cells were harvested after differentiation in induction medium (see above).

### Electrophoretic Mobility Shift Assay (EMSA)

Double-stranded DNA oligos corresponding to the sequences of the MIA/CD-RAP-dependent human *p54^nrb^* promoter region (5′- and 3′-hp54^nrb^, Table 1) and two double-stranded mutated DNA oligos corresponding to the 5′-hp54^nrb^ region (5′-hp54^nrb^ Mut I and 5′-hp54^nrb^ Mut II, Table 1) were synthesized by Sigma. The fragments correspond to the human *p54^nrb^* promoter regions from -7079 to -7062 (5′-hp54^nrb^) and from -7061 to -7038 (3′-hp54^nrb^) upstream of the ATG start site. The fragments were end labeled, and the EMSA assay was performed as previously described [27].

### Immunohistochemical staining

Paraffin sections of human and mouse cartilage tissues were screened for YBX1 protein expression by immunohistochemistry. Formalin-fixed and paraffin-embedded tissues were deparaffinized, rehydrated, heat treated with Tris–EDTA and subsequently incubated with primary YBX1 antibody (1∶20; #4202, Cell Signaling) for 30 min at room temperature. The sections were incubated with secondary antibody (human: Labeled Polymer, HRP - EnVision+; Dako, Carpinteria, CA, USA; mouse: Labeled Polymer, HRP - Histofine anti-rabbit; Nichirei Biosciences Inc., Tokyo, Japan) for 30 min at room temperature. Antibody binding was visualized using DAB+Substrate Chromogen solution (Dako). Finally, the tissues were counterstained with hematoxylin (Merck, Darmstadt, Germany).

### Bioinformatics analysis

HGU133PUS2 chip data, which were previously normalized by Dehne et al. (GSE16464), were used to identify differentially expressed genes between differentiated and undifferentiated chondrocytes. In total, 175 genes sharing the motif GATTGG in their promoter region were tested using a paired Student's t-test; 62 differentially expressed genes were identified (adjusted p value <0.05, Benjamini-Hochberg correction).

### Statistical analysis

The mean and standard deviation values were calculated from the independent experiments. Graphs were prepared using the GraphPad Prism® 4.03 software (GraphPad Software Inc., San Diego, CA, USA). Statistical significance between two groups was determined using Student's t-test in GraphPad Prism 4. Statistical significance was determined using data from at least three independent experiments. A p-value <0.05 was considered statistically significant and marked with an asterisk (ns: not significant, *p<0.05; **p<0.01; ***p<0.001).

## Results

We previously showed that p54^nrb^ acts as a mediator of MIA/CD-RAP action during cartilage development to promote chondrogenesis [7] and that there was evidence of transcriptional regulation of p54^nrb^ expression [12]. To analyze the regulatory mechanism underlying the regulation of the *p54^nrb^* gene by MIA/CD-RAP in chondrocytes, we analyzed a highly conserved region of the *p54^nrb^* promoter.

### *p54^nrb^* promoter regulation is MIA/CD-RAP dependent

**Figure 1:**
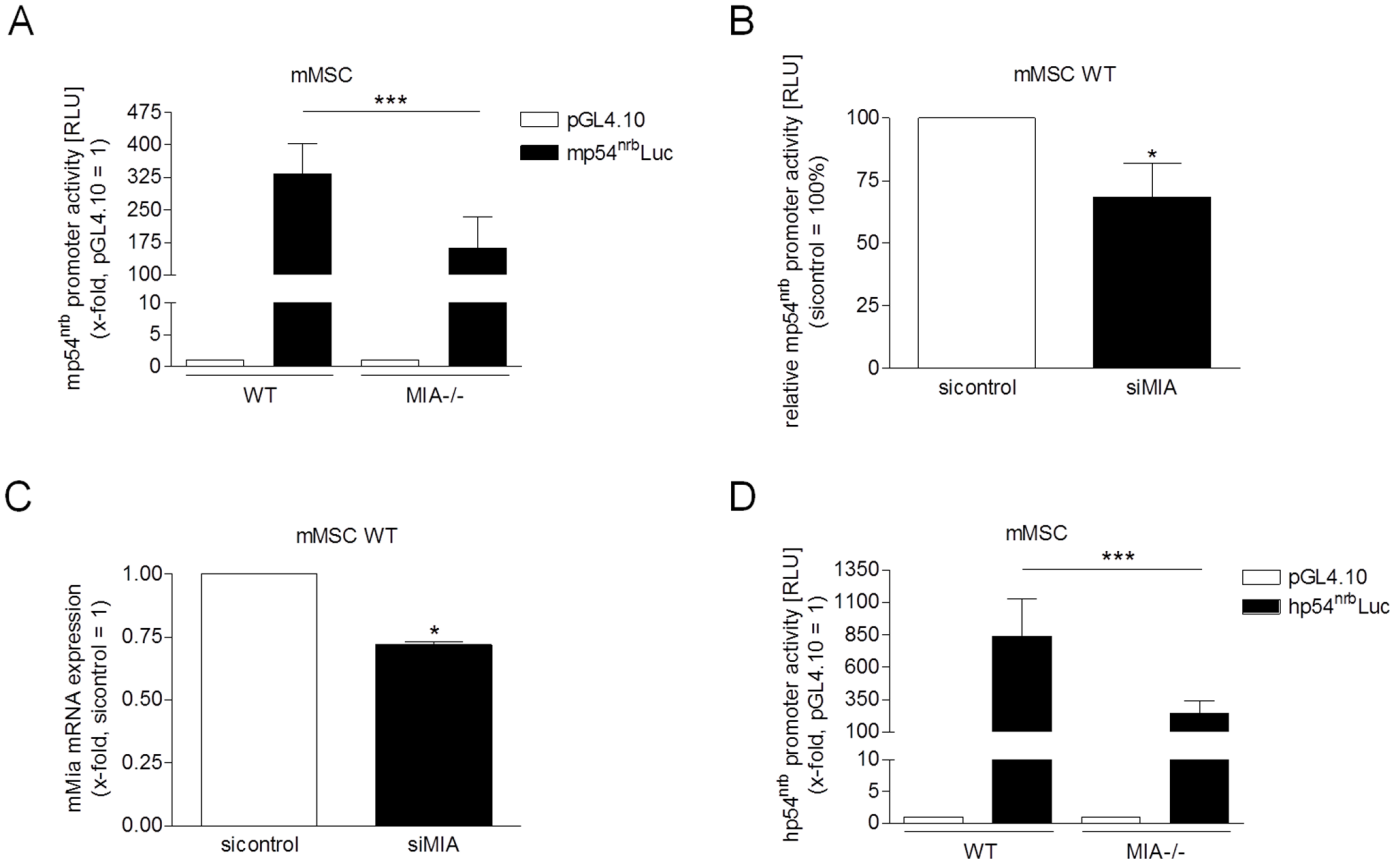
*p54^nrb^* promoter activity is controlled by MIA/CD-RAP. (**A**) Luciferase reporter assays revealed strong activity of the murine *p54^nrb^* (mp54^nrb^) promoter in primary murine mesenchymal stem cells (mMSCs). In MIA/CD-RAP-deficient (MIA-/-) mMSCs, the promoter activity was reduced (***p<0.001). The values represent the mean ± s.d. from four independent experiments. (**B**) Murine *p54^nrb^* promoter activity analyzed using reporter gene assays was decreased in the wild-type (WT) mMSCs transfected with siRNA against MIA/CD-RAP compared with the control (*p<0.05). The values represent the mean ± s.d. from three experiments. (**C**) The knockdown efficiency of MIA/CD-RAP in WT mMSCs was validated using qRT-PCR. The MIA/CD-RAP mRNA expression was successfully inhibited compared to the siControl-transfected cells (*p<0.05). The values represent the mean ± s.d. from three independent experiments. (**D**) The human *p54^nrb^* promoter analyzed using reporter gene assays was highly active in WT mMSCs and showed reduced activity in MIA-/- mMSCs (***p<0.001). The values represent the mean ± s.d. from eleven experiments.

The activities of the mouse and human *p54^nrb^* promoters and the influence of MIA/CD-RAP were analyzed using luciferase reporter assays. Luciferase measurement after transient transfection of the murine *p54^nrb^* (*mp54^nrb^*) promoter construct into differentiated primary murine mesenchymal stem cells (mMSCs) revealed strong activity in the MIA/CD-RAP wild type cells and a marked reduction in mMSCs derived from the MIA/CD-RAP-deficient (MIA-/-) mice (Figure 1A). Diminished *mp54^nrb^* promoter activity in the wild-type (WT) mMSCs treated with siRNA specific to MIA/CD-RAP confirmed these data (Figure 1B). The knockdown efficiency of MIA/CD-RAP in the WT mMSCs was confirmed using quantitative real-time PCR (qRT-PCR) (Figure 1C).

Transient transfection of the *hp54^nrb^* promoter construct into murine MSCs also resulted in strong activity, whereas the transcriptional activity was reduced in MIA-/- mMSCs (Figure 1D). In conclusion, the human and murine *p54^nrb^* promoter constructs were strongly conserved, and both were functional in the mouse systems. Importantly, their activity was modulated by MIA/CD-RAP.

### MIA dependency is linked to a 42 bp DNA element in the *p54^nrb^* promoter

**Figure 2:**
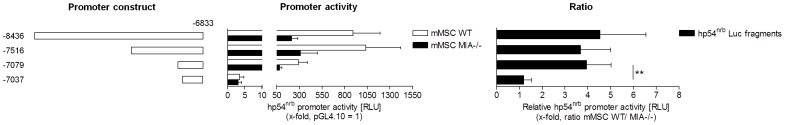
Mapping the *p54^nrb^* promoter region that mediates the effects of MIA/CD-RAP on *p54^nrb^* transcription. The human *p54^nrb^* promoter luciferase constructs ranging from residues −8436 to −7037 bp are indicated on the left. The luciferase activities resulting from transient transfections into WT (white bars) or MIA-/- mMSCs (black bars) are indicated in the middle. The ratios of human *p54^nrb^* promoter activities comparing WT and MIA-/- mMSCs are shown on the right for each luciferase construct. The ratio of the human *p54^nrb^* promoter activity comparing WT and MIA-/- mMSCs significantly decreased in deletions involving the region between −7079 and −7037 bp, identifying this 42 bp DNA region as the mediator of MIA/CD-RAP up-regulation of *p54^nrb^* promoter activity (*p<0.05). The values represent the mean ± s.d. from three experiments.

Based on a previous study in melanoma [15], we investigated a series of truncated *p54^nrb^* promoter-luciferase constructs. The defined human *p54^nrb^*-luciferase constructs were transfected into primary WT and MIA-/- mMSCs (Figure 2). Figure 2 shows a summary of the promoter constructs on the left and the resulting promoter activities in the center. On the right, the ratio comparing the WT and MIA-/- promoter activity is depicted, illustrating the influence of MIA/CD-RAP when the ratio is significantly higher than 1. We revealed that the promoter region between −7079 and −7037 bp is necessary and sufficient for MIA/CD-RAP to increase the *p54^nrb^* promoter activity in mesenchymal stem cells. In summary, these assays led to the conclusion that the 42 bp DNA element between −7079 and −7037 bp in the *p54^nrb^* promoter is highly conserved and mediates MIA/CD-RAP-induced gene transcription during chondrogenesis.

### Identification of transcription factor binding sites in the p54^nrb^ promoter

**Figure 3:**
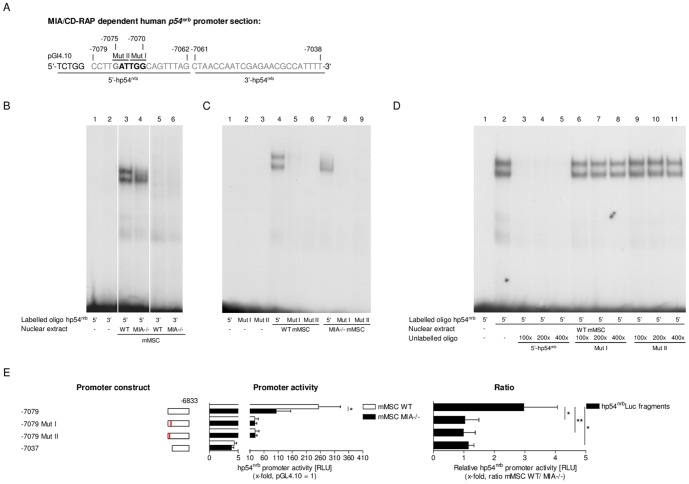
EMSA competition experiments and reporter gene assays demonstrated binding ability to one specific region in the *p54^nrb^* promoter. (A) Schematic overview of the MIA/CD-RAP-dependent region between −7079 and −7038 bp, highlighted in grey, of the human *p54^nrb^* promoter-luciferase reporter. The oligos used for the EMSA assay were labeled as follows: 5′-, 3′-hp54^nrb^ and 5′-hp54^nrb^ Mut I and II. The mutated regions are shown in bold letters. The numbers represent the locations with respect to the ATG translation start codon. (B) EMSA analysis revealed two strong DNA-protein complexes interacting with the 5′-hp54^nrb^ oligo in the nuclear extracts from mMSCs (lane 3). Binding capacity of MIA-/- mMSC (lane 4) nuclear extracts was diminished. No binding was observed with the 3′-hp54^nrb^ oligo (lane 5 and 6). (C) Incubation of nuclear extracts from WT mMSCs (lane 4) with the 5′-hp54^nrb^ oligo confirmed the formation of two strong DNA-protein complexes that were diminished when incubated with MIA-/- mMSC nuclear extracts (lane 7). Mutations I and II in the 5′-hp54^nrb^ oligo abolished the formation of DNA-protein complexes in the nuclear extracts from WT cells and MIA-/- mMSCs (lane 5, 6 and 8, 9). The contents of the reaction mixtures are marked in the tables below the images of the gels. (D) For competition experiments, the gel shifts were assessed in competition mixtures containing three different concentrations (x-fold) of unlabeled 5′-hp54^nrb^ oligo (lanes 3–5) or unlabeled mutated oligos (Mut I and II) (lanes 6–8 and 9–11, respectively). In contrast to incubation with unlabeled 5′-hp54^nrb^ oligo, incubation with the unlabeled mutated oligos did not lead to competition of the complexes formed by the binding of the labeled 5′-hp54^nrb^ oligo to WT mMSC nuclear extracts (lane 2). (E) On the left, human *p54^nrb^* promoter-luciferase constructs ranging from residues −7079 to −7037 are shown. Luciferase activities resulting from transient transfections are presented in the center. The ratios of human *p54^nrb^* promoter activities in the WT and MIA-/- mMSCs are shown to the right of each luciferase construct. Mutation of site I or II in the *p54^nrb^* promoter significantly abolished the MIA/CD-RAP-mediated *p54^nrb^* promoter activation as measured using the reporter gene assay (*p<0.05; **p<0.01). The values represent the mean ± s.d. from three independent experiments each.

Putative transcription factor binding sites within the genomic region between −7079 and −7037 bp of the human *p54^nrb^* promoter (Figure 3A) were defined using the Gene2Promoter program (www.genomatix.de). Two double-stranded oligomeric fragments corresponding to 18 bp of the 5′ region and to 24 bp of the 3′ region of the 42 bp DNA element (5′- and 3′-hp54^nrb^, Figure 3A) were used in the electrophoretic mobility shift assays (EMSA). We noted two strong DNA-protein complexes involving the 5′-hp54^nrb^ oligo using nuclear extracts from primary mMSCs; these complexes were reduced in the nuclear extracts from MIA-/- mMSCs (Figure 3B). Nuclear extracts from WT mMSCs showed no differential binding to the 3′-hp54^nrb^ oligo compared to the respective MIA-negative cells (Figure 3B). Using the MatInspector program (www.genomatix.de), we analyzed the genomic region between −7079 and −7037 bp to determine the locations of the specific regulatory elements. This analysis revealed two potential binding sites for several transcription factors located between −7075 and −7070 bp. We designed two double-stranded oligomeric fragments in which the first or the second binding site in this region was mutated (5′-hp54^nrb^ Mut I and II). The formation of a strong DNA-protein complex using nuclear extracts from WT mMSCs was observed in gel mobility shift assays, and these complexes were diminished when incubated with MIA-/- mMSC nuclear extracts (Figure 3C). Mutations of the transcription factor binding sites (5′-hp54^nrb^ Mut I and -II) nearly abolished the formation of these DNA-protein complexes (Figure 3C).

To confirm the binding of WT mMSC nuclear extracts to the 5′-hp54^nrb^ oligo, we performed competition experiments using three different ratios of unlabeled 5′-hp54^nrb^, -Mut I and -Mut II oligos in excess. Interestingly, even small amounts of unlabeled 5′-hp54^nrb^ oligo eliminated the DNA-protein interaction between the WT mMSC nuclear extracts and the 5′-hp54^nrb^ oligo. Furthermore, incubation with even the highest fold excess of unlabeled Mut I or Mut II oligo resulted in no reduction in complex formation between the WT mMSC nuclear extract and the 5′-hp54^nrb^ oligo, thus suggesting these oligos could not compete for binding (Figure 3D).

Next, we investigated the promoter activity of the −7079 to −6833 bp *p54^nrb^* promoter construct containing mutations I and II. The transfection of these mutated *p54^nrb^* promoter constructs into primary mMSCs revealed that both mutations led to an almost complete loss of MIA/CD-RAP-dependent *p54^nrb^* promoter activity, as measured by the reporter gene assay (Figure 3E, right graph illustrates ratio). In summary, the *p54^nrb^* promoter region from −7075 to −7070 bp relative to the ATG protein start codon is responsible for the MIA/CD-RAP-dependent activation of the *p54^nrb^* promoter during cartilage differentiation.

#### Identification of the transcription factor that activates the p54^nrb^ promoter in a MIA/CD-RAP-dependent fashion

**Figure 4:**
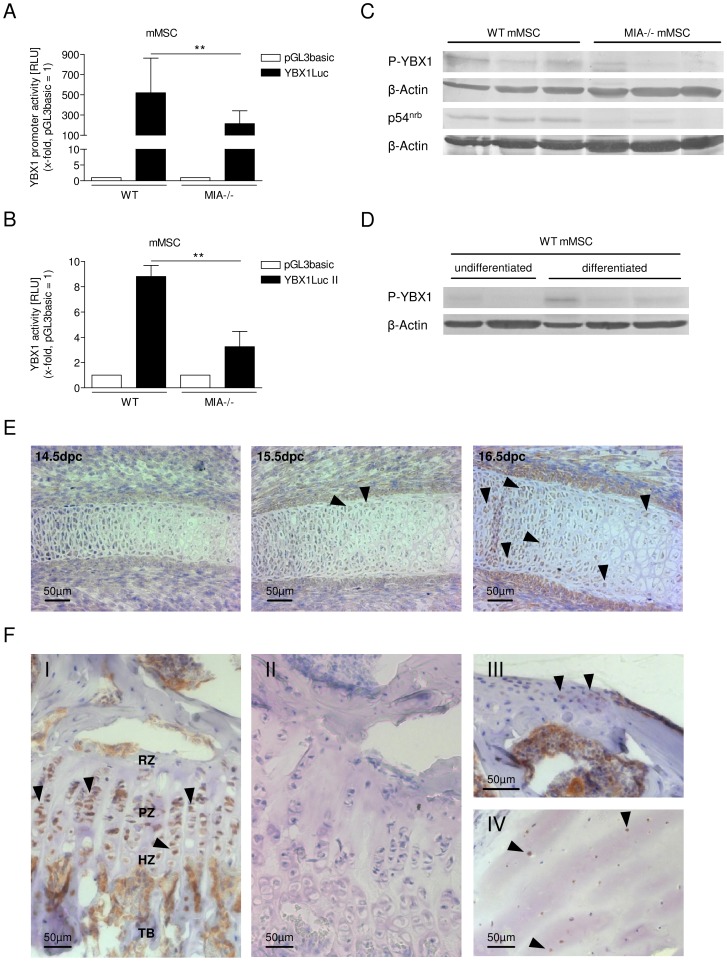
MIA/CD-RAP activates the *p54^nrb^* promoter via the transcription factor YBX1. (**A**) YBX1 activity as measured using a reporter gene assay revealed a significant decrease in YBX1 promoter activity in MIA-/- mMSCs compared with WT mMSCs (III) (**p<0.01). The values represent the mean ± s.d. from eight independent experiments. (**B**) Luciferase reporter assays revealed the significant inhibition of YBX1Luc II activity in MIA-/- mMSCs compared with WT mMSCs (**p<0.01). The values represent the mean ± s.d. from four experiments. (**C**) Detection of p54^nrb^ and phospho-S102 YBX1 protein levels in WT and MIA-/- mMSCs analyzed by western blot analysis. Consistent with the reduced p54^nrb^ protein levels, the levels of phospho-S102 YBX1 were also considerably reduced in the MIA/CD-RAP-negative cell system compared with the control. (**D**) Western blot analysis revealed elevated phospho-S102 YBX1 protein levels during differentiation of WT mMSC. (**E**) Immunohistochemical staining of YBX1 in rips of embryos at different developmental stages. At stage 14.5 days post coitum no YBX1 staining was detected. Scattered and slightly stained chondrocytes were assessed in ribs of 15.5dpc embryos. At 16.5dpc, strong YBX1 expression was detected in chondrocytes and hypertrophic chondrocytes. (**F**) Immunostaining of YBX1 in tibial growth plates revealed its strong expression in chondrocytes of the proliferative and hypertrophic chondrocyte zone and bone marrow (I). Positive signals were also detected in mouse (II) and human (II) cartilage. The black arrows mark positive stained cells. RZ: resting chondrocyte zone; PZ: proliferative chondrocyte zone; HZ: hypertrophic chondrocyte zone; TB: trabecular bone.

We used the MatInspector analysis to identify the MIA/CD-RAP-dependent transcription factor mediating the activation of the *p54^nrb^* promoter. In a recent study in melanoma, we found that YBX1 could transcriptionally regulate MIA/CD-RAP-dependent p54^nrb^ transcription [15]. Until today, no link between YBX1 and chondrogenesis has been observed; however, we aimed to define whether this regulation was also important in cartilage differentiation. Reporter gene assays using a YBX1 promoter luciferase construct (YBX1Luc) [23] revealed significantly lower YBX1 activity in MIA-/- differentiated mMSCs than in the WT differentiated mMSCs (Figure 4A). To examine this phenomenon in more detail, a luciferase reporter vector with four tandem repeats of the Y-box consensus oligo linked to a minimal promoter was used (YBX1Luc II) [24]. Consistent with the previous results, we showed a significant reduction in YBX1Luc II activity in the MIA-/- differentiated mMSCs compared with differentiated WT mMSCs (Figure 4B). The phosphorylation of YBX1 at serine 102 (S102) is required for its transcriptional activity [28]; we therefore analyzed the level of phospho-S102 YBX1 in a panel of differentiated WT and MIA-/- mMSCs (Figure 4C). P54^nrb^ protein levels served as controls. Together with the reduced p54^nrb^ protein levels, phospho-S102 YBX1 was considerably reduced in the MIA-/- mMSCs compared to the WT mMSCs. Ultimately, we defined YBX1 as the transcriptional activator of the *p54^nrb^* promoter that mediates the regulatory activity of MIA/CD-RAP in chondrogenesis.

### YBX1 in cartilage differentiation

The role of YBX1 in chondrogenic differentiation and in cartilage in general has not yet been addressed so far. For this reason, we performed differentiation experiments to determine the role of YBX1 in this process. We analysed the level of phospho-S102 YBX1 protein in a panel of undifferentiated and differentiated WT mMSCs. Phospho-S102 YBX1 was elevated in differentiated WT mMSC compared to undifferentiated cells (Figure 4D). To reveal the role of YBX1 during cartilage development *in vivo*, YBX1 expression was examined in embryonic cartilage tissue of C57BL/6 wild-type mice by immunostaining with an antibody raised against YBX1 (Figure 4E). In embryos at stage 15.5 days post coitum (dpc) scattered, slightly stained chondrocytes were detectable in the growth plate, whereas at 14.5dpc no YBX1 staining was found. At 16.5dpc, strong YBX1 staining was detected in chondrocytes and in some hypertrophic chondrocytes of the embryonic growth plate at embryonic stage 16.5dpc (Figure 4E). Immunostaining of adult murine cartilage tissue revealed intense YBX1 staining in chondrocytes of the proliferative and hypertrophic chondrocyte zone of the growth plate and in the bone marrow of SV/129 wild-type mice (Figure 4F I). No false positive staining was observed due to the detection system itself (Figure 4F II). These findings are in agreement with the protein expression of differentiated YBX1 in murine MSCs (Figure 4C). Further, we detected YBX1 expression in chondrocytes of mouse (SV/129 wild-type) and human articular cartilage (Figure 4F III+IV).

### Identification of the conserved sites in genes associated with cartilage differentiation

**Figure 5:**
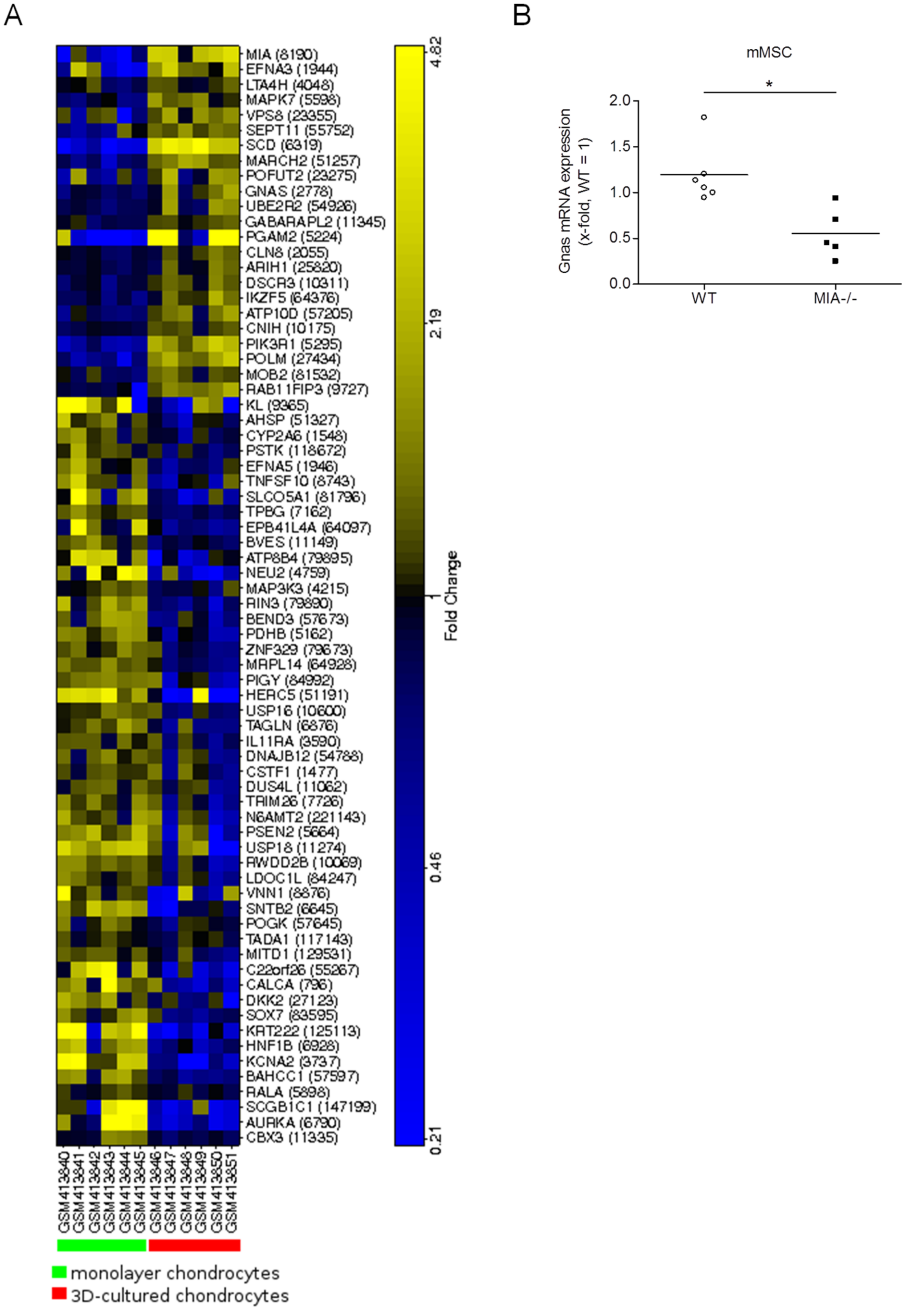
The YBX1 site is conserved in genes associated with cartilage differentiation. (**A**) Gene expression differences in the genes with promoters containing the regulatory region GATTGG, which spans the −7075 to −7070 bp region relative to the ATG in the *p54^nrb^* promoter region. Genes are distributed along the x-axis, and the chondrocytic samples are distributed along the y-axis (GSE16464). Chondrocyte differentiation correlates with MIA/CD-RAP expression levels [4], [3], [29]. The relative gene expression levels are color coded to indicate higher (yellow) and lower (blue) expression levels. The genes are distributed along the y-axis, and the cell data are distributed along the x-axis. (**B**) The mRNA expression levels of Gnas measured by qRT-PCR were significantly reduced in different lots of differentiated MIA-/- mMSCs compared with WT mMSCs The values represent the mean ± s.d. from five experiments. (*p<0.05).

To reveal further downstream effectors that mediate the effect of MIA/CD-RAP on cartilage differentiation, we screened the promoter regions of additional genes for the presence of the MIA/CD-RAP-dependent regulatory sequence found in the *p54^nrb^* promoter. A comprehensive scan of the human genome with the sequence TTGATTGGCAGT revealed the identities of 175 sequences in promoter regions of specific genes; these sites are located within 10 kb upstream of the annotated transcription start sites. We assumed that at least a subset of these genes is in fact regulated by MIA/CD-RAP-YBX1 during chondrocyte differentiation and, therefore, should show a strong correlation with cartilage differentiation and elevated MIA/CD-RAP expression [4], [3], [29]. To identify a set of potential YBX1 targets, we used the transcriptional profiles available at GSE16464. Of the 175 genes already identified as having an YBX1 binding site, 155 genes were found on the microarrays. A paired t-test was performed to identify significantly differentially expressed genes between the differentiated and undifferentiated chondrocytes (Figure 5A). We chose one of the differentially expressed genes (Gnas) known to be involved in chondrocyte differentiation [30] and confirmed the change in the mRNA expression levels of this gene in MIA/CD-RAP-negative differentiated mesenchymal stem cells (Figure 5B).

In summary, our results revealed the effects of secreted MIA/CD-RAP on the putative transcriptional regulation of additional genes via the YBX1 transcription factor, thereby establishing important connections between MIA/CD-RAP and new downstream effectors in cartilage development. Further, YBX1 was shown to be important in cartilage development for the first time.

## Discussion

MIA/CD-RAP is an important regulator of chondrogenesis; however, the signaling induced by MIA/CD-RAP, which is a secreted molecule, remains mostly unknown. We showed previously that p54^nrb^ acts as a mediator of MIA/CD-RAP action to promote chondrogenesis in its early stages by modulating proliferation and differentiation [7]. In this study, we therefore concentrated on the analysis of p54^nrb^ regulation by MIA/CD-RAP as a model system to understand MIA/CD-RAP molecular signaling and to determine its target genes in chondrogenesis.

In agreement with our study in melanoma cells [15], we revealed that MIA/CD-RAP-dependent modulation of the *p54^nrb^* promoter activity is controlled by a conserved 42 bp DNA element. It is very interesting that these two different cell types share this common mechanism of regulation. This can be seen as proof of conserved regulation following exposure to MIA/CD-RAP. The importance of YBX1 in the MIA/CD-RAP-dependent regulatory region of the *p54^nrb^* promoter should be confirmed in the chondrogenic system; however, until now, YBX1 was not known to play a role in chondrocyte differentiation or in cartilage development. Consistent with the binding of YBX1 to the promoter region, we revealed MIA/CD-RAP-dependent activation of YBX1 by S102 phosphorylation, confirming the activation of YBX1 in chondrocytes during differentiation. Here, we have made a major step in understanding the promotion of chondrogenesis by MIA/CD-RAP via its downstream target p54^nrb^.

YBX1 expression had not been implicated in chondrocyte differentiation or cartilage previously. There are some hints in the literature that YBX1 could be important in cartilage as YBX1-deficient mice die around the time that cartilage differentiation begins [21], [31]. YBX1 is also known to play a role in insulin signaling, which is important in cartilage differentiation [32]. However, its role in cartilage differentiation per se was not addressed in these publications. Here, we revealed the expression and activity of YBX1 in mMSCs in response to MIA/CD-RAP. Interestingly, immunohistochemistry clearly confirmed the impact of YBX1 in cartilage development. Because this expression pattern is similar to that of MIA/CD-RAP during cartilage development [4], [3], these findings underline the role of YBX1 as a mediator of MIA/CD-RAP in chondrocyte differentiation. To further uncover downstream effectors that may mediate the effects of MIA/CD-RAP during chondrogenesis via the regulation of YBX1, we screened for potential YBX1 binding sites in the promoters of genes associated with cartilage differentiation. We identified 62 genes with potential YBX1 binding sites and coherent expression across differentiated chondrocytes. The MIA/CD-RAP-dependent regulation of one of these genes (Gnas) was analyzed as a proof of concept and could be readily confirmed based on its mRNA levels in mesenchymal stem cells. Gnas encodes the α subunit (Gsα) of a stimulatory heterotrimeric G protein (Gs) that transduces signals from various cell-surface receptors to adenylyl cyclases. Gsα was shown to be the primary mediator of the actions of the PTH/PTHrP receptor in growth plate chondrocytes, as Gnas-deficient chondrocytes undergo premature hypertrophy [30]. Therefore, the activation of YBX1 through MIA/CD-RAP is a new aspect in the understanding of how MIA/CD-RAP fulfills its role in chondrogenesis.

YBX1 activation and nuclear translocation is mainly controlled by the phosphorylation of S102 on YBX1 via the PI3K/AKT pathway [33], [28]. In a previous study, we showed that in chondrocytes, ERK signaling is inhibited by MIA/CD-RAP via its binding to integrin α5, which in turn inhibits its activity [34]. However, in mesenchymal stem cells, the inhibition of ERK signaling did not lead to the regulation of p54^nrb^ expression at either the mRNA or the protein level [7]. Hence, the intracellular signaling pathway that underlies the activation of p54^nrb^ expression by MIA/CD-RAP and YBX1 remains unclear, as no other MIA/CD-RAP-regulated signaling pathways have been described to date.

In summary, we showed that the transcription factor YBX1 is activated by MIA/CD-RAP and is necessary for the activation of the *p54^nrb^* promoter, providing insight into that activity of MIA/CD-RAP during chondrogenesis. The discovery of a new, previously unknown function of YBX1 in cartilage and the identification of its targets provide new avenues of research to understand how MIA/CD-RAP fulfills its role in chondrogenic differentiation.
